# Evidence summary on management strategies for gastroesophageal reflux symptoms in patients following esophageal cancer surgery

**DOI:** 10.1016/j.apjon.2024.100639

**Published:** 2024-12-10

**Authors:** Yuqing Zhao, Yaxin Fu, Wei Zhang, Shengjiang Zhao, Huixia Li

**Affiliations:** a Esophageal Oncology Department, Tianjin Medical University Cancer Institute & Hospital, National Clinical Research Center for Cancer, Tianjin’s Clinical Research Center for Cancer, Key Laboratory of Cancer Prevention and Therapy, Tianjin, China; b School of Nursing, Tianjin Medical University, Tianjin, China; c School of Nursing, Tianjin University of Traditional Chinese Medicine, Tianjin, China; d Nursing Department, Tianjin Second People’s Hospital, Tianjin, China

**Keywords:** Gastroesophageal reflux symptoms, Self-management, Esophageal cancer, Evidence-based practice, Post-surgical care

## Abstract

**Objective:**

This study aimed to summarize evidence-based strategies for the self-management of gastroesophageal reflux symptoms (GERS) at home among patients who have undergone esophageal cancer surgery, providing practical references for clinical practice.

**Methods:**

A systematic evidence summary was conducted based on the reporting standards of the Fudan University Center for Evidence-based Nursing. Literature was retrieved from international and Chinese databases, including guidelines, expert consensus, systematic reviews, and original studies. The search covered the period from the inception of the databases to June 30, 2024. Two independent reviewers appraised the quality of evidence and synthesized recommendations across six domains: reflux symptoms, assessment, treatment, health education, follow-up, and outcome indicators.

**Results:**

A total of 25 high-quality studies were included, comprising 10 guidelines, 10 expert consensus documents, two systematic reviews, and three original studies. Thirty-four evidence items were synthesized, emphasizing a combination of pharmacological treatments, lifestyle modifications, and health education to improve self-management outcomes.

**Conclusions:**

This evidence synthesis highlights effective strategies for home-based self-management of GERS after esophageal cancer surgery. Future research should focus on culturally tailored interventions and large-scale studies to further enhance the applicability and reliability of these findings.

**Trial registration:**

This study was registered at the Fudan University Center for Evidence-Based Nursing (Registration No. ES202446701).

## Introduction

Recent studies have^1^ indicated that esophageal cancer ranks eleventh in incidence among all cancers and seventh in terms of mortality rate.^2^ Digestive tract reconstruction is necessary following intraoperative esophagectomy,^3^ with most surgeons opting for a tubular stomach in place of the esophagus.^4^^,^^5^ However, replacing the esophagus with a tubular stomach can disrupt the %normal anti-reflux mechanism, resulting in a high (81%) incidence of gastroesophageal reflux after the surgery.^6^

Gastroesophageal reflux disease (GERD) is characterized by a range of symptoms, end-organ effects, and complications caused by the reflux of gastric and duodenal contents into the esophagus, oral cavity (including the throat), and/or lungs.^7^ Following esophageal cancer surgery, patients with a residual esophagus may experience organic damage due to prolonged exposure to the reflux fluid.^8^^,^^9^ In severe cases, this condition can lead to aspiration pneumonia, acute respiratory distress syndrome, and septic shock due to the aspiration of refluxed materials.^10^^,^^11^ According to relevant surveys,^12^ gastroesophageal reflux is the only symptom that does not improve with prolonged recovery time after esophageal cancer surgery. Clinical experts recommend that patients should undergo long-term home management of esophageal reflux symptoms after esophageal cancer surgery.^13^

The relevant guidelines clearly indicated that the treatment of gastroesophageal reflux necessitates a combination of lifestyle interventions and pharmacological therapy for effective management.^14^^,^^15^ Self-management is a strategy that harnesses an individual’s intrinsic motivation to facilitate behavioral changes.^16^ It has been established that implementing home-based self-management techniques for reflux symptoms after esophageal cancer surgery is crucial for controlling symptoms. For example, in a study enrolling patients who underwent esophageal cancer surgery, elevating the head of the bed led to 46.1% reduction in the severity of esophagitis, while 38.5% of patients demonstrated stabilization after three months. This finding suggests that effective self-management practices can significantly improve the reflux symptoms of patients in a home setting.^17^^,^^18^

While interventions for gastroesophageal reflux symptoms have been documented in both domestic and international studies, the quality of these interventions varies significantly.^19^ Currently, there is a lack of systematic and comprehensive content addressing the symptoms of esophageal reflux in patients who have undergone surgery for esophageal cancer. Furthermore, there is a dearth of guiding research on relevant self-management practices in this context.^20^

In times of multimedia, it is challenging for nurse practitioners to obtain reliable evidence and guide clinical decision-making amidst a vast amount of information.^21^^,^^22^ Additionally, the standardization, systematization, and facilitation of self-management practices for gastroesophageal reflux symptoms in–home patients after surgery for esophageal cancer is a critical area requiring further exploration.^23^ In this study, we systematically searched for research on the self-management of gastroesophageal reflux symptoms. We aimed to summarize the best available evidence regarding the self-management of gastroesophageal reflux symptoms in–home patients undergoing surgery for esophageal cancer. This was achieved through a systematic and comprehensive search, followed by a rigorous evaluation to establish a reliable foundation for clinical nursing practice.

## Methods

### Question identification

The PIPOST model was utilized to formulate evidence implementation questions.^24^ The components of the model were as follows: (i) Population (P): home patients and caregivers following esophageal cancer surgery; (ii) Intervention (I): measures related to the management of reflux symptoms, which include screening assessments, treatment care, and follow-up; (iii) Professional (P): health care workers; (iv) Outcome (O): enhancement of knowledge and self-management behaviors regarding reflux symptoms among patients and their families at home after esophageal cancer surgery; (v) Setting (S): specialist wards, families, and communities; (vi) Type of evidence (T): clinical decision-making,guidelines, evidence summaries, best practices, and recommendations.

### Literature retrieval strategy

Based on the ‘6S’ pyramid model,^25^ top-down searches were conducted across computerized decision systems, guideline websites, professional association websites, and related databases. The systematic searches of computerized decision systems included BMJ Best Practiceand Up To Date. Guideline websites comprised the World Health Organization (WHO), the Guidelines International Network (GIN), the National Institute for Health and Clinical Excellence (NICE), and the National Guideline Clearinghouse (NGC). Professional association websites featured the Registered Nurses Association of Ontario (RNAO) and the European Society of Intensive Care Medicine (ESICM). Evidence-based databases included theCochrane Library and the Joanna Briggs Institute (JBI), while comprehensive databases comprised CINAHL, Web of Science, PubMed, Cochrane Library, and Embase. Additionally, the Chinese databases were reviewed including the China Biomedical Literature Service System (SinoMed), WanFang Database, Chinese Journal Full-textDatabase (CNKI), and the VIP Chinese Science and Technology Journal Database (VIP). The search period extended from the inception of the database until August 21, 2023, with updates made on June 30,2024.

**Fig. 1:**
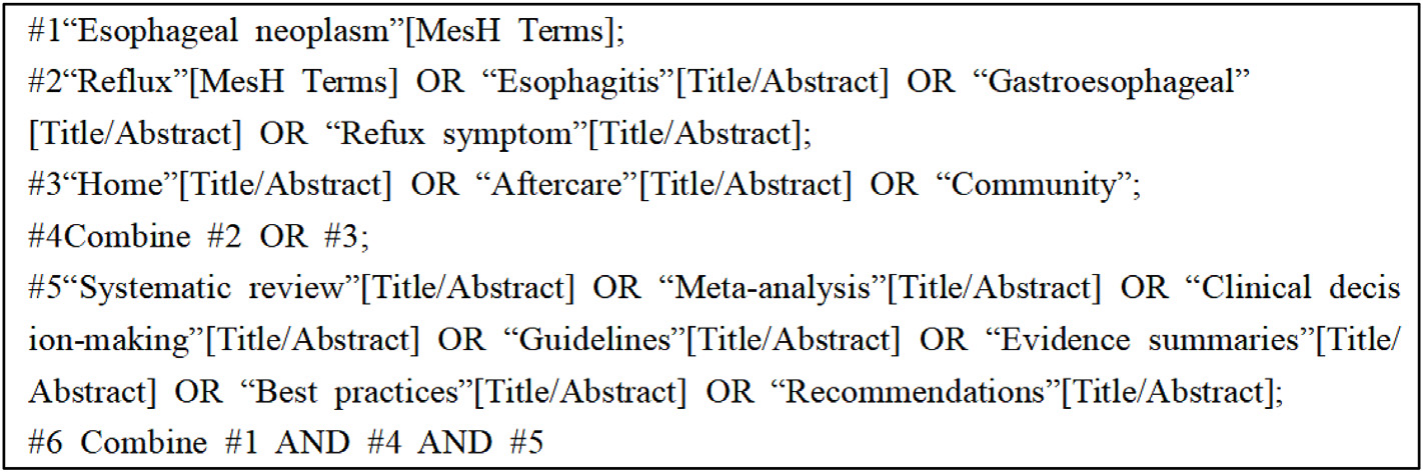
PubMed search strategy (modify).

An example of an English database search using PubMed with the corresponding search strategy is shown in Fig. 1.

### Literature inclusion and exclusion criteria

For included studies, the participants were necessarily home patients undergoing surgery for esophageal cancer, with a focus on the measures of self-management for gastroesophageal reflux symptoms. The inclusion criteria were as follows: (i) study subjects consisted of patients and caregivers who were 18 yearsor older and underwent treatment for esophageal cancer; (ii) the study involved standardized management protocols for addressing home reflux symptoms after treating esophageal cancer; (iii) outcome indicators included the knowledge and behaviors of patients and caregivers regarding home reflux symptoms and reflux-related outcome measures; (iv) the type of literature included guidelines, evidence summaries, expert consensus, clinical decisions, and systematic evaluations; (v) The literature was available in both Chinese and English. The exclusion criteria were as follows: (i) documents without full text; (ii) studies that were republished by multiple organizations or had a more recent version; (iii) non-English and Chinese literature.

### Literature quality evaluation

The guidelines were evaluated using the Appraisal of Guidelines for Research and Evaluation (AGREE II) tool.^26^ Clinical decision-making, evidence summaries, best practices, and recommended practices were assessed through the original literature tracing method, with the appropriate Joanna Briggs Institute (JBI) evaluation tool selected based on the type of literature.^27^ Both expert consensus and systematic reviews were assessed using a tool from the Australian JBI Evidence-Based Health Care Center.^28^

All researchers underwent systematic training in evidence-based methodologies. Four researchers independently assessed the guidelines, and the intraclass correlation coefficient (ICC) was employed to measure the consistency of the results.^29^ In cases of discrepancy, an authoritative expert in the relevant field adjudicated the findings. The remaining studies were independently evaluated by two researchers, with a third researcher resolving any disagreements.

### Evidence summary and recommendation level

In conjunction with the outcomes of the previous literature retrieval, two researchers established a comprehensive framework that details the evidence and content within each dimension. They meticulously classified, extracted, and summarized relevant evidence. The original level of evidence was documented asoriginating from guidelines. In instances where conflicting evidence originated from various sources, we applied the principle of prioritizing high-grade, high-quality, and newly published evidence.^30^ The JBI Evidence Pre-Grading and Evidence Recommendation System was employed for evidence originating from systematic reviews, expert consensus, and preliminary studies lacking assigned evidence levels.^31^ This system utilizes a scale of 1–5, with level 1 denoting the highest quality and level 5 denoting the lowest quality. Subsequently, the evidence was classified into grade A (strongly recommended) and grade B (weakly recommended) based on the JBI feasibility, appropriateness, meaningfulness, and effectiveness (FAME) structure.^32^

## Results

### Baseline characteristics of the enrolled articles

**Fig. 2:**
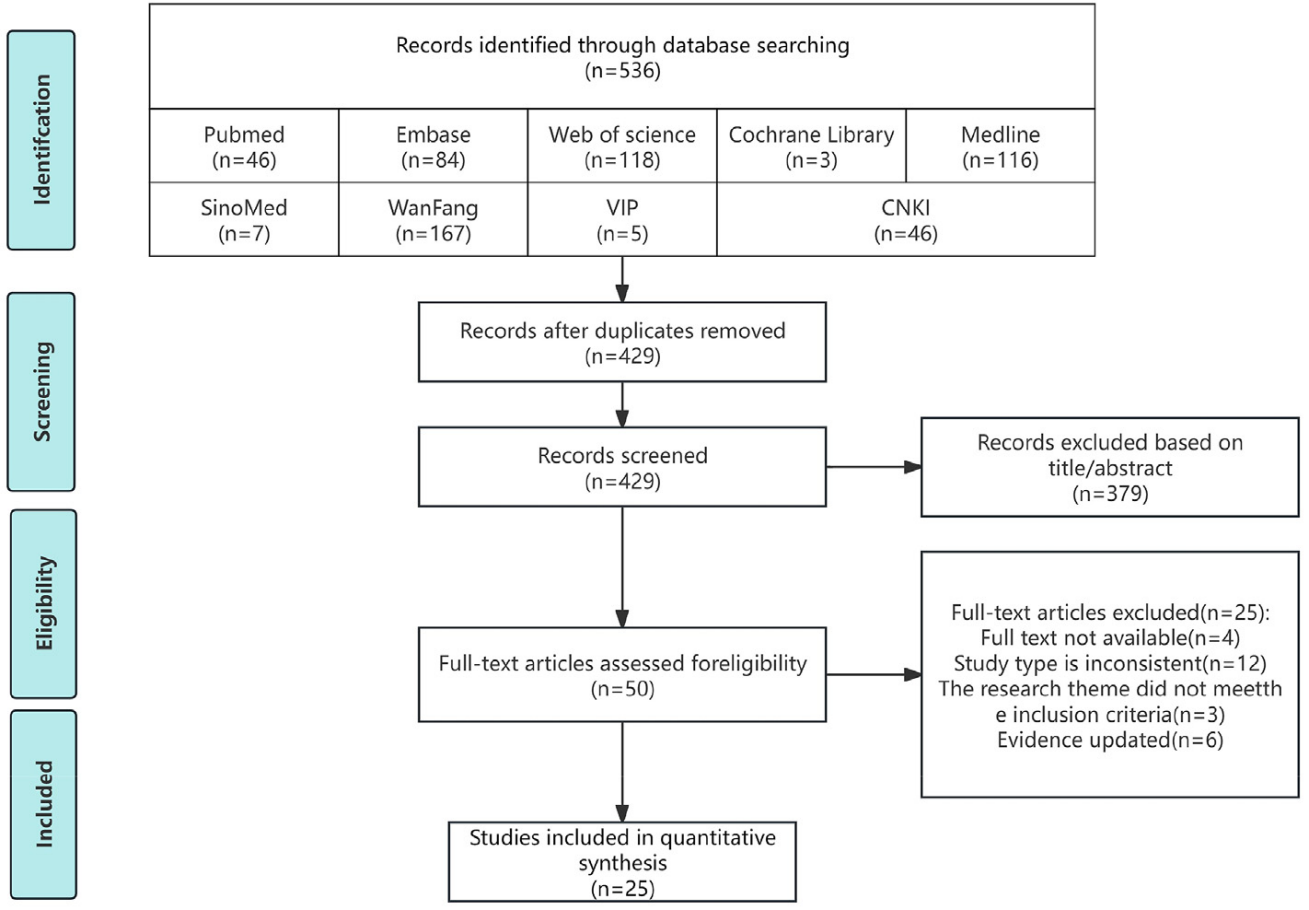
Screening ﬂow chart (modify).

Fig. 2 presents the PRISMA^33^ flow diagram that illustrates the literature retrieval process. Initially, 536 records were obtained. After the removal of duplicates, 429 records remained. Following a review of the titles and abstracts, 379 records were excluded, leading to the selection of 50 studies for full-text screening. Finally, 25 articles were eligible for inclusion, consisting of 10 guidelines, 10 expert consensus articles, three original studies, and two systematic reviews (Table 1).

**Table 1:** Characteristics of the included articles (*N* ​= ​25).

Author (year)	Article type	Article source	Research subject
Hunt R et al.^34^(2015)	Clinical practice guidelines	Medical pulse	Management of gastroesophageal reflux disease
Kahrilas PJ et al.^35^(2016)	Clinical practice guidelines	Medical pulse	Chronic cough caused by patients with GERD
CMA^36^(2016)	Clinical practice guidelines	WanFang database	Grassroots diagnosis and treatment of gastroesophageal reflux disease
Hunt R et al.^37^(2017)	Clinical practice guidelines	PubMed	A global perspective of gastroesophageal reflux disease
Seo HS et al.^38^(2018)	Clinical practice guidelines	Medical pulse	Surgical treatment of gastroesophageal reflux disease
Groulx S et al.^39^(2020)	Clinical practice guidelines	Medical pulse	Screening for esophageal adenocarcinoma in patients with chronic gastroesophageal reflux disease
Lwakiri K et al.^40^(2021)	Clinical practice guidelines	Medical pulse	Treatment of gastroesophageal reflux disease
Jung HK et al.^14^(2021)	Clinical practice guidelines	Medical pulse	Diagnosis and treatment of gastroesophageal reflux disease
Katz PO et al.^41^(2022)	Clinical practice guidelines	PubMed	Diagnosis and management of gastroesophageal reflux disease
Slater BJ et al.^42^(2023)	Clinical practice guidelines	Medical pulse	Treatment of gastroesophageal reflux disease
Moraes-Filho J et al.^43^(2002)	Specialist consensus	PubMed	Evaluation, classification, and management recommendations of gastroesophageal reflux disease
Pace F et al.^44^(2009)	Specialist consensus	PubMed	Definition and classification of gastroesophageal reflux disease
Fock KM et al.^45^(2016)	Specialist consensus	Medical pulse	Management of the gastroesophageal reflux disease
Zhang Shengsheng et al.^46^(2017)	Specialist consensus	Medical pulse	TCM diagnosis and treatment of gastroesophageal reflux disease
Gyawali CP et al.^47^(2018)	Specialist consensus	Medical pulse	Diagnosis of gastroesophageal reflux disease
CGS^48^(2020)	Specialist consensus	WanFang database	Diagnosis and treatment of gastroesophageal reflux disease in China
Zerbib F et al.^49^(2021)	Specialist consensus	Medical pulse	Diagnosis and treatment of refractory gastroesophageal reflux disease
CSGERD^50^(2021)	Specialist consensus	WanFang database	Endoscopic treatment of gastroesophageal reflux disease
CCMIP^51^(2022)	Specialist consensus	Medical pulse	Multidisciplinary diagnosis and treatment of gastroesophageal reflux disease
CCMIP^52^(2022)	Specialist consensus	Medical pulse	Multidisciplinary diagnosis and treatment of gastroesophageal reflux disease
Casale M et al.^53^(2016)	Systems assessment	PubMed	Effect of lower esophageal sphincter respiratory training on gastroesophageal reflux disease
Ness-Jensen E et al.^54^(2016)	Systems assessment	PubMed	Lifestyle intervention for gastroesophageal reflux disease
He Haisheng et al.^55^(2005)	Randomized controlled trial	CNKI	Prevention and management of anastomotic reflux after cervical anastomosis in esophageal cancer
Zhuang Jun et al.^56^(2008)	Randomized controlled trial	CNKI	Nursing care of gastroesophageal reflux after esophageal cancer
Li ZY et al.^57^(2023)	Qualitative study	PubMed	Supportive care needs of patients discharged after esophageal cancer

### Results of literature quality evaluation

In total, 10 guidelines were included.^14^^,^^34^, ^35^, ^36^, ^37^, ^38^, ^39^, ^40^, ^41^, ^42^ Based on the percentage of normalized scores across various fields, namely, scope, and purpose, stakeholder involvement, rigor of development, clarity of presentation, applicability, and editorial independence—the guidelines discussed by Hunt R et al.^34^^,^^36^^,^^37^^,^^40^^,^^42^ received a grade B recommendation. In contrast, the remaining five guidelines received scores of ≥ 60% across the six criteria, resulting in a grade A recommendation. The results of the quality evaluation are presented in Table 2. Ten expert consensus articles were included.^43^, ^44^, ^45^, ^46^, ^47^, ^48^, ^49^, ^50^, ^51^, ^52^ Based on the percentage of normalized scores in each field, which included view source, expert influence, population interests, statement conclusion, reference, and opinion heterogeneity, four expert consensus articles received a grade B recommendation, while six expert consensus articles received an A grade. Additionally, two systematic reviews were included,^53^^,^^54^ with one receiving a B grade and the other an A grade recommendation. Furthermore, three clinical practice articles were included.^55^, ^56^, ^57^ Two randomized controlled trials (RCTS) received grade B recommendations, while a qualitative study received an A grade.

**Table 2:** Quality evaluation results of guidelines (*N* ​= ​10).

Included in the literature	The percentage of normalized scores in each field（%）						60% of fields (a)	30% of fields (s)	Recommend rank
	Scope and purpose	Participant	Preciseness	Clarity	Practicability	Independent character			
Hunt R^34^	91.67%	86.11%	80.21%	88.89%	58.33%	83.33%	5	0	B
Kahrilas PJ^35^	100.00%	100.00%	91.67%	100.00%	93.75%	100.00%	6	0	A
Chinese Medical Association^36^	97.22%	100.00%	29.17%	83.33%	68.75%	70.83%	5	1	B
Hunt R^37^	100.00%	77.78%	37.50%	97.22%	75.00%	54.17%	4	0	B
Seo HS^38^	100.00%	100.00%	91.67%	100.00%	93.75%	100.00%	6	0	A
Groulx S^39^	97.22%	97.22%	95.83%	94.44%	89.58%	95.83%	6	0	A
Lwakiri K^40^	91.67%	88.89%	52.08%	86.11%	85.42%	79.17%	5	0	B
Jung HK^14^	97.22%	88.89%	80.21%	86.11%	83.33%	95.83%	6	0	A
Katz PO^41^	94.44%	69.44%	78.13%	94.44%	89.58%	95.83%	6	0	A
Slater BJ^42^	94.44%	100.00%	55.21%	75.00%	77.08%	79.17%	5	0	B

### Evidence description and summary

A comprehensive integration of the related content was conducted after the extraction, analysis, comparison, and discussion of the collected evidence. This meticulous process led to the synthesis of 32 pieces of compelling evidence, summarized across six aspects: reflux symptoms, assessment, treatment, health education, follow-up procedures, and outcome indicators (Table 3).

**Table 3:** Evidence summary.

Evidence item	Evidence content	Evidence level	Recommendation grade
Reflux symptoms and diagnosis	1. Core symptoms: Heartburn (burning sensation after the sternum: Heartburn generally occurs 30–60 min after eating), acid reflux/reflux (acid content returns to the mouth), and increased saliva (excessive saliva secretion)^14^^,^^34^^,^^36^, ^37^, ^38^^,^^40^, ^41^, ^42^, ^43^, ^44^^,^^48^^,^^50^^,^^51^^,^^57^	5	A
	2. Alarm symptoms: Belching (belching), burning sensation in the upper abdomen, upper abdominal pain, abdominal pain, chest pain (preheart area), dysphagia, slow digestion, early satiety, nausea, vomiting, respiratory symptoms (cough, wheezing, chronic sinusitis), ear, nose, and throat symptoms (hoarseness, sore throat)^14^^,^^34^^,^^36^, ^37^, ^38^^,^^43^, ^44^, ^45^^,^^48^^,^^50^^,^^51^	5	A
	3. Diagnosis: At least twice a week for 4 or 8 weeks or more of heartburn or reflux symptoms indicate gastroesophageal reflux symptoms^34^^,^^43^	5	B
	4. On normal endoscopy, the 24-h esophageal pH ​< ​4 suggests gastric reflux^37^^,^^40^^,^^41^^,^^47^^,^^48^^,^^52^^,^^54^	5	A
	5. If symptoms do not relieve, or patients are older than 40 years and have alarm symptoms (dysphagia, anemia, gastrointestinal bleeding, and weight loss), esophagogastroduodenoscopy (EGD) should be conducted, in which mucosal erosion (esophagitis) or digestive stricture is diagnosed as gastroesophageal reflux disease^39^^,^^40^^,^^41^^,^^43^^,^^47^^,^^52^^,^^54^	5	A
Assessment	6. Timing of medication assessment: Assessment before drug use; PPI medication 8 for 12 weeks and then evaluation^34^^,^^36^^,^^39^	5	A
	7. Symptoms: The incidence, severity, and frequency of heartburn and reflux and alarm symptoms should be recorded from 1 day before discharge to 12 weeks after discharge^34^^,^^36^^,^^37^	5	B
	8. Indisposing factors: Eating time, type of diet (proportion of fat content), activity (whether you often bend over) and bed rest (posture and time) from 1 day before discharge to 12 weeks after discharge^34^^,^^36^^,^^37^^,^^43^	5	B
	9. Mitigating factors: Sodium bicarbonate, antacids, milk, OTC drugs (antacids or alginate-antacids) from 1 day before discharge to 12 weeks after discharge^34^^,^^37^^,^^43^	5	B
Pharmacological interventions	10. Medications: Antacids, H2 receptor antagonists, and proton pump inhibitor (PPI) should be prescribed for 4 weeks^36^^,^^40^^,^^41^^,^^48^^,^^54^	5	A
	11. Side effects of PPIs include headache, abdominal pain, nausea, vomiting, diarrhea, constipation, and flatulence. In case of side effects, stop using the prescribed drugs^41^	5	A
	12. The treatment plan, the use of treatment drugs, and the possible adverse reactions should be thoroughly introduced to patients, to encourage them to receive sufficient treatment and prevent reducing or stopping drugs^36^	5	B
	13. PPIs should be taken 30–60 minutes before breakfast and dinner^37^^,^^49^	5	B
	14. Avoiding drugs that reduce LES pressure and affect gastric emptying, such as nitroglycerin, anticholinergic drugs, theophylline, calcium channel blockers, etc.^36^	5	B
Non-Pharma-cological interventions	15. Smoking is strictly prohibited (quit smoking) and drinking is restricted^36^^,^^41^^,^^48^^,^^38^^,^^54^	5	A
	16. Keeping doing physical exercise^54^	5	B
	17.Obese patients should undergo weight loss and keep their BMI at < 25 kg/m^2^^14^^,^^35^^,^^36^^,^^41^^,^^45^^,^^48^, ^49^, ^50^	5	A
	18. Food types: Avoid foods that may induce reflux attacks and heartburn, such as coffee, chocolate, mint, citrus, carbonated drinks, high-fat foods, and spicy foods, and eat more foods rich in dietary fibers^34^^,^^36^^,^^37^^,^^46^	5	A
	19. Eating time: avoid meals 2–3 hours before going to bed, and avoid midnight snacks^34^^,^^36^^,^^41^^,^^44^	5	A
	20. Diet times: eat less and more meals^34^^,^^37^^,^^41^	5	A
	21. Exercising after eating: After each meal, let the patient be in the upright or upright position for 0.5 h, walk, and promote food emptying with the help of gravity; avoid the movement of bending down after eating^57^	3	A
	22. Bed head elevation: Use a sleep positioning device (pillow), and raise the bedside by 15–20° to avoid regurgitation before and after meals^42^^,^^46^^,^^48^^,^^49^^,^^54^	5	A
	23. Left lateral decubitus position^49^	5	A
	24. Respiratory exercise: IMT inspiratory muscle training (five days per week for two months)/seven days per week, 8 weeks, once in the morning and evening; the 1st and 2nd exercises focus on supine abdominal breathing, moving abdominal wall, combating resistance, and relaxing thoracic and subcostal muscle groups. The 3rd, 4th and 5 exercises focus on sitting and standing inhalation training, exhaling slowly, following abdominal movements, and raising arm sound^53^	1	B
	25. Psychological guidance: GERD is characterized by chronic delay and recurrence of the disease, which can easily increase the ideological burden of patients and decrease compliance with medical behavior. Through active communication, address patients' concerns and psychological obstacles, and enhance the confidence to overcome the disease^36^^,^^46^	5	B
	26. Abdominal massage: fry heat with fennel, wrap cloth on the abdomen, and massage around^56^	1	B
	27. Acupressure massage: 30–60 min after meals, Neiguan and Zhongwan, and each acupoint pressure should be 5∼10min^56^	1	B
Follow-up procedures	28. Frequency of follow-up: once every 2–4 weeks until reaching the standard (the standard is the disappearance of clinical symptoms and no reflux esophagitis on auxiliary examination); frequency of reaching standard follow-up: once every 3 months^36^	5	B
	29. Follow-up and evaluation: Comprehensive assessment of medical history, symptom recurrence, response to anti-acid therapy, lifestyle improvement, and physical examination, including blood pressure, heart rate, heart rhythm, height, weight, waist circumference, and auxiliary examination. Endoscopy should be conducted when necessary to evaluate the risk of GERD and the clinical situation, which can help determine the treatment strategy^36^	5	B
	30. Annual assessment: Establishing consultation platforms or WeChat groups to facilitate peer group activities. In addition to the above-mentioned follow-up items conducted every 3 months, endoscopy is feasible to assess the condition if necessary^36^^,^^54^	5	B
Outcome indicators	31. Esophageal quality of life Questionnaire, EQOL: Used to evaluate the quality of life of potentially curable esophageal cancer patients, the score ranges from 0 to 100, with higher scores indicating better quality of life. This study sets the standard for achievement to be above 60 points.^55^	1	B
	32. Gastroesophageal reflux disease questionnaire (Gerd-Q): Symptoms within 1 week before patient presentation.This scale is used to detect the severity of gastroesophageal reflux symptoms, with a score range of 0–8 points. In this study, the standard for compliance was set to below 8 points.^14^^,^^34^^,^^36^^,^^34^^,^^46^^,^^48^^,^^51^	5	A

## Discussion

This evidence summary is the first to provide a comprehensive overview of home-based self-management strategies for reflux symptoms following esophageal cancer surgery. It encompasses six key aspects: reflux symptoms and diagnosis, assessment, pharmacological interventions, non-pharmacological interventions, follow-up procedures, and outcome indicators. Therefore, this study summarized the best evidence for the self-management of gastroesophageal reflux symptoms for the family members of patients undergoing esophageal cancer surgery, thereby providing a reliable basis for the development and implementation of home management solutions.

### Reflux symptoms and diagnosis

Evidence 1–9 summarizes the reflux symptoms of patients after esophageal surgery and describes the assessment tools. The reflux symptoms experienced by these patients differ from those associated with typical gastroesophageal reflux due to the loss of the esophageal sphincter after esophageal cancer surgery. Furthermore, reflux symptoms may manifest atypically as increased saliva production and hoarseness. Thus, this study provided a description of related symptoms identified through a literature search, an aspect that was overlooked by previous studies addressing the self-management of gastroesophageal reflux symptoms following esophageal cancer surgery.^58^ After surgical recovery, it is essential for patients to have a comprehensive understanding of the methods for assessing reflux symptoms before engaging in self-management at home. This understanding should encompass the ability to easily determine the timing of symptoms, inducing factors, mitigation strategies, and monitoring techniques. Numerous studies have demonstrated that patient self-assessment and recording at home can enhance their awareness and effectiveness of self-management.^59^^,^^60^ Sunjaya AP *et al.*^61^ reported the benefits of utilizing remote medical assistance to assess the relevant symptoms of patients. Therefore, based on this study, health care professionals are encouraged to employ Internet technology to strengthen guidance for assessing reflux symptoms at home.

### Assessment

Evidence 10–14 summarizes the therapeutic measures available for patients with gastroesophageal reflux symptoms, encompassing both pharmacological interventions. Although many patients respond favorably to medication, some may not experience significant benefits. Our study indicated that functional exercise improves reflux symptoms.^53^ The diaphragm plays a crucial role in the physiological anti-GERD barrier, inducing a 3-fold–4-fold increase in pressure within the gastroesophageal junction (GEJ) region. In clinical practice, we observed postoperative reflux among patients with esophageal cancer and they were more dependent on drugs, while the implementation of non-pharmacological interventions has not received enough attention. Therefore, medical staff should guide patients to provide guidance on symptom management before discharge and recommend follow-up support after discharge to reduce the incidence of reflux.

### Pharmacological interventions

Evidence 15–27 summarizes the health education needed for the self-management of gastroesophageal reflux symptoms in patients undergoing surgery for esophageal cancer. Effective lifestyle management is a critical component of self-care for patients with esophageal cancer who undergo surgery. Authoritative evidence indicates that drug therapy may lead to certain side effects;^62^ therefore, medical professionals should guide patients to establish appropriate daily activities and engage in self-management at home. International studies have underscored the importance of implementing various lifestyle measures to prevent reflux, including smoking cessation, avoidance of alcohol, regular physical exercise, weight control, and avoiding foods that may trigger reflux. Several studies have suggested that professional and consistent home-based health guidance is essential for encouraging patients to adhere to a healthy lifestyle and engage in long-term self-management. Despite the current emphasis on home guidance, limitations in medical resources often hinder physicians from providing adequate support. Related studies have confirmed that nurses play a pivotal role in delivering home guidance. By offering specialized advice for reflux-preventive lifestyles through accessible methods, nurses can enhance patients' self-management capabilities, enhance their confidence, and improve their self-management behavior.

### Follow-up procedures and outcome indicators

Evidence 28–32 summarizes the follow-up procedures and outcome indicators for patients experiencing gastroesophageal reflux symptoms. This study found that patients who underwent esophageal cancer surgery were followed every 2–4 weeks. They were followed based on a comprehensive history assessment until all health indicators met the established standards. Each follow-up session focused on detecting symptoms and patients' responses to self-management. Additionally, attention should be paid to caregiver follow-up visits and sharing self-management experiences among peers. Due to the lack of effective means for communication with hospital nurses when facing difficulties in self-management, corresponding internet tools should be developed to address patients' consultation needs, thereby facilitating effective follow-up. The center utilized WeChat to provide a small program platform for reflux management. It was employed for patient consultations, nurse follow-ups, and outcome index evaluations. Since the majority of patients with esophageal cancer are old, out-of-hospital follow-up is crucial for promoting healthy aging within the framework of continuous care in China. Therefore, based on a systematic literature review, we proposed a structured follow-up process to improve patients' self-management ability at home. In conclusion, caregivers should prioritize monitoring patients' adherence to prescribed behaviors during the post-discharge follow-up phase. Furthermore, an appropriate assessment scale should be selected based on the individual conditions of the patients to effectively monitor their outcomes.

### Implications for nursing practice and research

The retrieval strategy was meticulously formulated with an evidence-based strategy through comprehensive discussions, ensuring a thorough search across various databases. The team’s involvement in the evaluation, classification, and summarization of evidence enhanced the reliability of our findings. In light of the scarcity of literature regarding self-management of reflux symptoms at home following esophageal cancer surgery, this study systematically integrated high-quality evidence in this field, offering valuable insights for medical professionals involved in esophageal cancer treatment and nursing.

### Limitations

However, this study also had some limitations. It exclusively included studies in Chinese and English, which might have resulted in the omission of relevant evidence from other languages. Furthermore, the systematic synthesis of domestic and international studies might not fully capture the realities of diverse cultural contexts. Therefore, clinical nursing staff should judiciously select and apply evidence based on local conditions and the specific circumstances of patients during clinical practice.

## Conclusions

This study employed evidence-based methods to summarize best practices for home-based self-management after esophageal cancer surgery, concentrating on six key areas: reflux symptoms, assessment, treatment, health education, follow-up procedures, and outcome indicators. This comprehensive review provides invaluable evidence for home-based self-management of gastroesophageal reflux symptoms after esophageal cancer surgery. Although existing evidence supports the self-management of gastroesophageal reflux symptoms in patients after the treatment of esophageal cancer, the overall quality of evidence remains limited. To address this gap, future studies should prioritize high-quality translational and practice studies. Multi-center studies with larger sample sizes can improve the management of patients with gastroesophageal reflux. This approach can provide more robust and reliable evidence, finally strengthening the postoperative self-management of gastroesophageal reflux after esophageal cancer surgery and improving the scientific rigor and effectiveness of clinical practice.

## CRediT authorship contribution statement

**Yuqing Zhao:** Conceptualization, Methodology, Data curation, Formal analysis, Writing. **Yaxin Fu:** Methodology, Writing - Original draft preparation. **Wei Zhang:** Formal analysis, Writing - Revised draft preparation, Data curation. **Shengjiang Zhao:** Writing - Original draft preparation. **Huixia Li:** Conceptualization, Resources, Writing – review & Editing, Project administration. All authors had full access to all the data in the study, and the corresponding author had final responsibility for the decision to submit for publication. The corresponding author attests that all listed authors meet authorship criteria and that no others meeting the criteria have been omitted.

## Ethics statement

The study was approved by the Ethics Committee of the Cancer Hospital of Tianjin Medical University (IRB No. bc2023199).

## Funding

This work was supported by the Tianjin Key Medical Discipline (Specialty) Construction Project (Grant No. TJYXZDXK-011A) in China. The funders played no role in the study design, data collection, analysis, interpretation, writing of the report, or the decision to submit the article for publication.

## Data availability statement

The data that support the findings of this study are available from the corresponding author, Huixia Li, upon reasonable request.

## Declaration of generative AI and AI-assisted technologies in the writing process

No AI tools/services were used during the preparation of this work.

## Declaration of competing interest

The authors declare no conflict of interest.
